# Gut Microbiome and Metabolome Were Altered and Strongly Associated With Platelet Count in Adult Patients With Primary Immune Thrombocytopenia

**DOI:** 10.3389/fmicb.2020.01550

**Published:** 2020-07-08

**Authors:** Xuewu Zhang, Silan Gu, Liangshun You, Yu Xu, De Zhou, Yunbo Chen, Ren Yan, Huiyong Jiang, Yating Li, Longxian Lv, Wenbin Qian

**Affiliations:** ^1^Department of Hematology, The First Affiliated Hospital, College of Medicine, Zhejiang University, Hangzhou, China; ^2^Key Laboratory of Hematopoietic Malignancies in Zhejiang Province, Hangzhou, China; ^3^Institute of Hematology, Zhejiang University, Hangzhou, China; ^4^State Key Laboratory for Diagnosis and Treatment of Infectious Diseases, National Clinical Research Center for Infectious Diseases, Collaborative Innovation Center for Diagnosis and Treatment of Infectious Diseases, The First Affiliated Hospital, College of Medicine, Zhejiang University, Hangzhou, China; ^5^Department of Hematology, The Second Affiliated Hospital, School of Medicine, Zhejiang University, Hangzhou, China

**Keywords:** immune thrombocytopenia, gut microbiota, metabolome biomarker, 16S rRNA gene sequencing, dysbiosis

## Abstract

Gut microbiota has been implicated in the pathogenesis of many autoimmune diseases. This is still an area of active research given that the role of gut microbiota on the primary immune thrombocytopenia (ITP) remains unclear. In this study, fecal samples of 30 untreated adult primary ITP patients and 29 healthy controls (HCs) were used to investigate the gut microbial community and metabolite profiles. Our results show that fecal bacteria such as *Blautia*, *Streptococcus*, and *Lactobacillus* are enriched, whereas bacteria such as *Bacteroides* are depleted in ITP patients. Notably, fecal metabolites such as fatty acyls and glycerophospholipids are enriched and strongly correlate with discrepant gut microbiota. Furthermore, combinations of *Weissella* and *Streptococcus anginosus*, or Cer (t18:0/16:0), Cer (d18:1/17:0), and 13-hydroxyoctadecanoic acid could provide good diagnostic markers for ITP. Moreover, a strong negative correlation was found between platelet count and altered gut microbiota such as *S. anginosus* and gut metabolites such as Cer (t18:0/16:0) in ITP. In conclusion, dysbiosis of both gut microbiota and metabolome develops in ITP patients compared to HCs. Several ITP-altered gut bacteria and metabolites can be diagnostic biomarkers for ITP, and are highly correlated with platelet count, suggesting that they may also play a role in ITP pathogenesis.

## Introduction

Primary immune thrombocytopenia (ITP), formerly known as idiopathic thrombocytopenic purpura, is an autoimmune disorder characterized by peripheral blood platelet count of less than 100 × 10^9^/L excluding conditions known to cause thrombocytopenia, such as infections, other autoimmune disorders, and drug effects (Rodeghiero et al., 2009). The incidence of ITP ranges from 16 to 27 new cases per million, while its prevalence ranges from 45 to 105 per million in adults and nearly 46 per million in children. At least two-thirds of children with ITP recover spontaneously within 6 months, but a relapse is common in adult patients following current standard first-line therapy with corticosteroids (Perera and Garrido, 2016).

Complex multifactorial alterations have been reported in the immune systems of patients with ITP, such as pathologic antiplatelet antibodies (McMillan, 2009; Webster et al., 2006), impaired platelet production (Khodadi et al., 2016), and T cell–mediated effects (Olsson et al., 2003). However, pathogenesis and etiology of ITP are still not fully understood. Genetic and environmental factors play an important role during this process, in a similar fashion to other autoimmune diseases. First, molecular mimicry that has been implicated in pathogenesis of many other autoimmune disorders may also contribute to occurrence of secondary ITP, such as between the glycoprotein 120 of human immunodeficiency virus or the core envelope 1 protein of hepatitis C virus and the platelet GPIIb/IIIa (Bettaieb et al., 1992; Zhang et al., 2009). Nevertheless, mechanisms other than molecular mimicry may dominantly contribute to secondary ITP. For instance, *Helicobacter pylori* decreases expression of the inhibitory receptor FcγRIIb on monocytes (Kodama et al., 2007). Secondly, ITP was reported to be improved by transplantation of fecal microbiota (Borody et al., 2011). Taken together, this suggests a potential role of gut microbiota in the pathogenesis of primary ITP.

The gut microbiota of many autoimmune diseases was altered and even was identified as one of the etiology. More than 1000 species of bacteria colonize the human gut, and play important roles in health and diseases (Tremaroli and Bäckhed, 2012). Nearly 60% of human immunity comes from the gut. The immune system of germ-free mice is underdeveloped, and their immune cells greatly decline. Recent studies have suggested a link between compositional and functional alterations of gut microbiota and disease manifestations, severity, and responsiveness to treatment (Proal and Marshall, 2018; Russell et al., 2019). Importantly, probiotic supplementation or fecal microbiome transplantation has been shown to be clinically beneficial to patients with autoimmune diseases (Xu et al., 2015; Uusitalo et al., 2016; Kouchaki et al., 2017). In addition, gut microbiota have also been associated with many extraintestinal autoimmune diseases and immune disorders, including rheumatoid arthritis, type 1 diabetes, multiple sclerosis, and system lupus erythematosus (Alkanani et al., 2015; Mu et al., 2015; Jangi et al., 2016; Maeda and Takeda, 2017). However, to the best of our knowledge, there has been no report on the role played by gut microbiota in primary ITP.

In the present study, we analyzed changes of 16S rRNA gene sequencing and liquid chromatography-mass spectrometry (LC-MS) based metabolomics in gut microbiota and fecal metabolic phenotype in patients to explore the gut microbiota characteristics and their role in adult primary ITP.

## Materials and Methods

### Study Participants

All primary ITP patients, used in the study, were diagnosed according to the established criteria (Lambert and Gernsheimer, 2017) and did not have other serious diseases or health problems. Stool samples were collected before therapy. Exclusion criteria for both ITP subjects and healthy controls (HCs) were as follows: no antibiotic use in the prior 6 months; no probiotic use; pregnancy or breast-feeding; history of gastrointestinal and cardiovascular diseases, diabetes, metabolic syndrome. Individuals who had a history of bowel surgery, irritable bowel syndrome, or other autoimmune disease were also excluded. Baseline characteristics of all participants are shown in Table 1. The study was approved by the ethics committee of the First Affiliated Hospital of Zhejiang University (reference number: 2018-42). Personal data and stool samples were collected according to the ethical guidelines of the Declaration of Helsinki. All participants signed an informed consent form before being enrolled in the study.

**TABLE 1:** Clinical features of ITP patients and HCs in this study.

	ITP (*n* = 30)	HCs (*n* = 29)	*P*-values
Age (years)	49.83 ± 2.46	49.55 ± 3.37	0.846
Sex (M/F)	9/21	10/19	0.713
BMI (kg/m^2^)	22.85 ± 2.42	23.28 ± 1.90	0.452
Basophil count (×10^9^/l)	0.016 ± 0.003	0.065 ± 0.031	2.51*E* − 4
Hemoglobin (g/l)	130.96 ± 3.82	141.44 ± 2.85	3.20*E* − 2
Platelet count (×10^9^/l)	13 ± 2.50	232.20 ± 9.69	4.19*E* − 11
Total protein (g/l)	67.77 ± 1.12	74.18 ± 0.64	1.00*E* − 5
Albumin (g/l)	43.89 ± 0.74	47.98 ± 0.49	2.90*E* − 5
Globulin (g/l)	23.85 ± 1.13	26.26 ± 0.51	4.40*E* − 2
ALT (U/l)	36.50 ± 6.41	20.48 ± 2.09	3.00*E* − 2
AST (U/l)	27.76 ± 3.13	19.24 ± 1.20	4.90*E* − 2
Direct bilirubin (mg/dl)	2.77 ± 0.23	4.14 ± 0.46	1.80*E* − 2
Creatinine (μmol/L)	55.33 ± 2.49	72.00 ± 2.79	8.91*E* − 5

*ITP, immune thrombocytopenia; HCs, healthy controls; BMI, body mass index; ALT, alanine aminotransferase; AST, aspartate aminotransferase.*

### DNA Extraction and Sequencing of 16S rRNA Gene

Sequencing of the 16S rRNA was performed as previously described (Lv et al., 2016). Briefly, bacterial genomic DNA was extracted using the QIAamp Fast DNA Stool Mini Kit (Qiagen, Hilden, Germany), according to the manufacturer’s instructions. The 16S rRNA V3–V4 region was amplified via PCR, and then sequenced using the MiSeq platform (Illumina, San Diego, CA, United States). The raw sequences are available on the NCBI Sequence Read Archive (SRA) database (Accession No. SRP191565).

### Sequence Analysis

Paired-end 16S rRNA sequencing data were joined and filtered using FLASH v1.2.10 software, and then chimeric sequences identified and removed using UCHIME (v4.2.40). All sequences were analyzed using QIIME (v1.9.1^1^), then UPARSE^2^ used to perform minor modifications and cluster high-quality sequences into operational taxonomic units (OTUs), using 97% similarity as a cutoff. The taxonomy of each 16S rRNA gene sequence was identified and classified by comparing them to those in the Silva (SSU123) 16S rRNA database with the Ribosomal Database Project (RDP) classifier algorithm^3^ using a 70% confidence threshold.

Bacterial richness and diversity among the samples were calculated using various indices, such as Chao 1 and Shannon. Differences in the composition of OTUs among different samples were analyzed using principal coordinates analysis (PCoA) plots implemented in R package^4^.

### Sample Preparation for Metabolic Profiling

To prepare samples for profiling metabolites, 20 μL of 2-chloro-l-phenylalanine (0.3 mg/mL) was first dissolved in methanol to prepare an internal standard. One milliliter of a methanol: water (7: 3 = v: v) mixture was then added to a 60 mg fecal sample and vortexed for 15 s, and left to stand for 5 min. Thereafter, the mixture was shaken for another 15 min and centrifuged for 15 min at 13,000 rpm. The supernatant was collected, filtered through a 0.22-μm microfilter, and pretreated for LC-MS analysis. Aliquots of all samples were pooled for quality control.

### LC-MS Analysis

An ACQUITY UHPLC system (Waters Corp., Milford, MA, United States), coupled with an AB SCIEX Triple TOF 5600 System (AB SCIEX, Framingham, MA, United States) were used to analyze the metabolic profiles in both electrospray ionization (ESI) positive and negative ion modes with an ACQUITYTM UPLC BEH C18 column (2.1 × 100 mm, 1.7 μm). The mobile phase consisted of solvents A water (containing 0.1% formic acid, v/v) and B acetonitrile (containing 0.1% formic acid, v/v). Each 5 μL sample was injected for separation using an elution gradient as follows: 5% B for 0 min; 20% B for 2 min; 25% B for 4 min; 60% B for 9 min; 100% B for 17 min; 100% B for 19 min; 5% B for 19 min; and 5% B for 20 min. The process involved a flow rate of 0.4 ml/min, and a column temperature of 45°C.

Data were captured in full scan (m/z ranging from 70 to 1000) combined with Information Dependent Acquisition (IDA) modes. The parameters for mass spectrometry were set as follows: ion source temperature, 550°C (+) and 550°C (−); ion spray voltage, 5500 V (+) and 4500 V (−); curtain gas of 35 PSI; de-clustering potential of 100 V (+) and −100 V (−); collision energy, 10 eV (+) and −10 eV (−); and interface heater temperature, 550°C (+) and 600°C (−). For IDA analysis, mass spectra were acquired over the mass to charge ratio range of 50–1000 m/z, and the collision energy was 30 eV.

### Multivariate Analysis of LC-MS Data

Raw LC-MS data, which are available in the “Baidu Netdisk^5^” with extraction code 2tw3, were captured with the progqenesis QI software (Waters Corporation) before analysis. Positive and negative datasets were integrated and analyzed using the SIMCA-P 14.0 software (Umetrics, Umea, Sweden). Metabolic alterations between the two groups were analyzed using principle component analysis (PCA) and orthogonal partial least squares discriminant analysis (OPLS-DA). Variable importance in the projection (VIP) was used to rank the overall contribution of each variable to the OPLS-DA model, and those variables with VIP > 1 were considered relevant for discriminating HC and ITP patients. Metabolites were identified by progqenesis QI Data Processing Software, based on public databases, including http://www.hmdb.ca/, http://www.lipidmaps.org/ and self-built ones.

### Statistical Analysis

The Mann–Whitney U test was used to compare any two data sets that were abnormally distributed for the clinical measurements [represented as mean ± standard deviation (SD)] as well as alpha diversity of the microbiota and metabolites; otherwise, a one-way analysis of variance (ANOVA) followed by the Student–Newman–Keuls method were performed. Beta diversity was assessed using analysis of similarities (ANOSIM), while the crucial bacterial taxa [represented as mean ± standard error of mean (SEM)] in the two groups were compared using the Wilcoxon rank sum test combined with the Benjamini–Hochberg method (Benjamini and Hochberg, 1995). The relationships between microbiota and metabolites were analyzed using the Spearman’s rank correlation test. In addition, the receiver operating characteristic (ROC) curve and area under curve (AUC) were calculated, using the pROC package, to assess diagnostic performance of the model. All data analysis procedures were performed using the SPSS statistical package (version 22.0) and R software (version 3.4.4). A *p*-value or *q*-value (false discovery rate adjusted) of less than 0.05 (*p* ≤ 0.05) were considered statistically significant.

## Results

### Characteristics of Participants

A total of 46 adult patients were diagnosed with primary ITP, with 16 meeting the exclusion criteria. The remaining 30 were included in the study. Twenty-nine HCs matched for age and sex were also enrolled. Platelet counts, as well as levels of hemoglobin, total protein, globulin, direct bilirubin and creatinine were lower, while those of alanine aminotransferase (ALT) and alanine aminotransferase (AST) were higher in ITP than HCs patients (Table 1).

### Differences in Gut Microbiota Between ITP Patients and HCs

**FIGURE 1:**
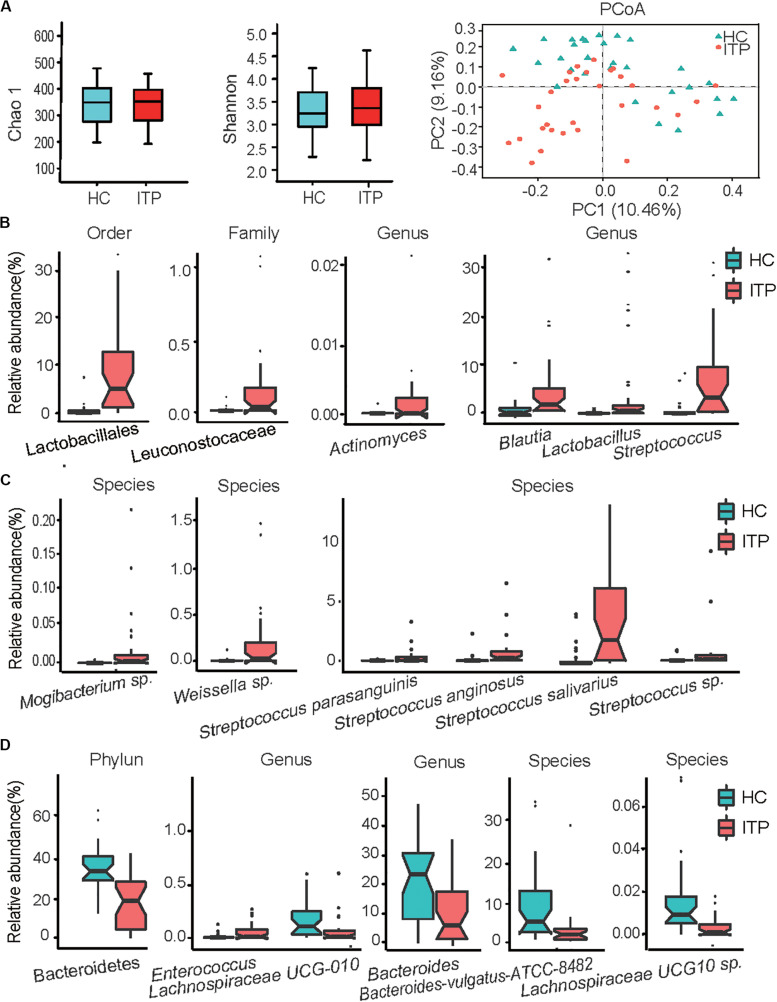
Alterations of gut microbiota in patients with primary immune thrombocytopenia (ITP) vs healthy controls (HCs). **(A)** Box plot of the Chao1 index, Shannon index and a two-dimensional plot of principal co-ordinates analysis built based on a bray matrix of the two groups. **(B)** Bacterial order, family, and genera enriched in feces of ITP patients. **(C)** Species enriched in ITP patients. **(D)** Phylum, genera and species depleted in ITP patients. The boxes represent the interquartile range, from the first and third quartiles, and the median is shown as a thick line in the middle of the box. The circles represent outliers beyond the whiskers. Each point in the graph represents a sample, and a color indicates a group of samples. *p*_ajust_ < 0.05 for all data selected.

From 59 fecal samples, we obtained 4,071,185 sequences following metagenomic sequencing of the V3–V4 region of the 16S rRNA gene. The number of sequences for these samples ranged from 54,487 to 74,946. During identification and classification, about 17 phylum, and 624 species with relative abundances ranging from 10^–7^ to 10^–1^, were observed. *Firmicutes, Bacteroidetes, Proteobacteria*, and *Actinobacteria* were the four most predominant phyla, accounting for more than 96% of the total sequences. In the alpha diversity analysis, neither the bacterial community richness (estimated by the Chao1 index) nor the community diversity (characterized by the Shannon index) was significantly different between the groups (Figure 1A). In beta diversity analysis, PCoA plots at the OTU level showed a separate clustering in microbiota profiles between the ITP and HCs (Figure 1A), with a significant difference (*r* = 0.14, *p* = 0.001) in composition of gut microbiota generated by ANOSIM.

Among *Firmicutes*, Lactobacillales enrichment (relative abundance, ITP vs HCs, 11.23 ± 2.21% vs 1.11 ± 0.43%, *p*_ajust_ = 4.29E-06) (Figure 1B) and its nine members was the main characteristic of alterations in gut microbiota in patients with ITP relative to HCs. The detectable rate of *Lactobacillus* genera was similar between the two groups, but a 30-fold increase in mean relative abundance (4.37 ± 1.74% vs 0.14 ± 0.06%, *p*_ajust_ = 5.34E-03) was recorded between HCs and ITP patients. Secondly, four species belonging to *Streptococcus* (6.49 ± 1.38% vs 0.89 ± 0.40%, *p*_ajust_ = 3.17E-04), including *Streptococcus anginosus, Streptococcus* sp., *Streptococcus parasanguinis* and *Streptococcus salivarius*, were enriched in ITP individuals (Figure 1C). Patients with ITP displayed a 35-fold overabundance of *S. anginosus* (mean ± SD, 0.36 ± 0.14%) compared to HCs (0.01 ± 0.01%, *p*_ajust_ = 1.51E-04). Furthermore, the detection rate of *S. anginosus* increased from 69% for HCs to 93% in ITP patients. Meanwhile, that of *Streptococcus* sp. (0.77 ± 0.39% vs 0.08 ± 0.05%, *p*_ajust_ = 1.51E-04) increased from 82 to 100%, an eightfold higher abundance in ITP patients. *S. parasanguinis* (0.85 ± 0.29% vs 0.16 ± 0.09%, *p*_ajust_ = 1.86E-03) and *S. salivarius* (4.50 ± 0.92% vs 0.59 ± 0.24%, *p*_ajust_ = 7.25E-04) were detected in all participants, with a 5–10-fold increase in mean relative abundances in the ITP group. Thirdly, although the mean relative abundance of *Enterococcus* in the two groups was similar, their detection rate increased from 44% for HCs to 80% in ITP patients. Additionally, between HCs and ITP patients, the detection rates of *Leuconostocaceae* (0.20 ± 0.06% vs 0.01 ± 0.004%, *p*_ajust_ = 1.30E-03) (Figure 1B) and its member *Weissella* sp. (0.19 ± 0.06% vs 0.01 ± 0.004%, *p*_ajust_ = 1.78E-03) (Figure 1C) increased from 40 to 80%, while their mean relative abundances increased by nearly 20-fold.

Another important alteration of gut microbiota in ITP patients was the depletion of Bacteroidetes (relative abundance, ITP vs HCs, 20.21 ± 2.75% vs 39.49% ± 2.49%, *p*_ajust_ = 3.24E-04) (Figure 1D). In this phylum, significantly depleted taxa included *Bacteroides* (10.22 ± 1.82% vs 21.89 ± 2.63%, *p*_ajust_ = 4.01E-02) and *Bacteroides vulgatus* ATCC8482 (2.83 ± 1.02% vs 9.39 ± 1.90%, *p*_ajust_ = 3.52E-02). In addition, the ratio of Firmicutes to Bacteroidetes, which is considered an important indicator of gut microbiota health, was significantly increased in the ITP group (32.03 ± 14.91% vs 1.58 ± 0.21%, *p* = 3.7E-05).

We observed an enrichment of low-abundance bacterial taxa, such as *Actinomycetaceae* (2.4 E-05 ± 1E-05 vs 2E-06 ± 8.87E-07, *p*_ajust_ = 4.45E-02) (Figure 1B) in ITP patients. However, *Lachnospiraceae UCG-010* (0.07 ± 0.03% vs 0.13 ± 0.03%, *p*_ajust_ = 4.21E-02) and *Lachnospiraceae UCG-010* sp. (3.4E-05 ± 9E-06 vs 2E-04 ± 4E-05, *p*_ajust_ = 1.05E-02) (Figure 1D) were depleted in ITP patients.

### Variations in Fecal Metabolomes in ITP Patients and HCs

**FIGURE 2:**
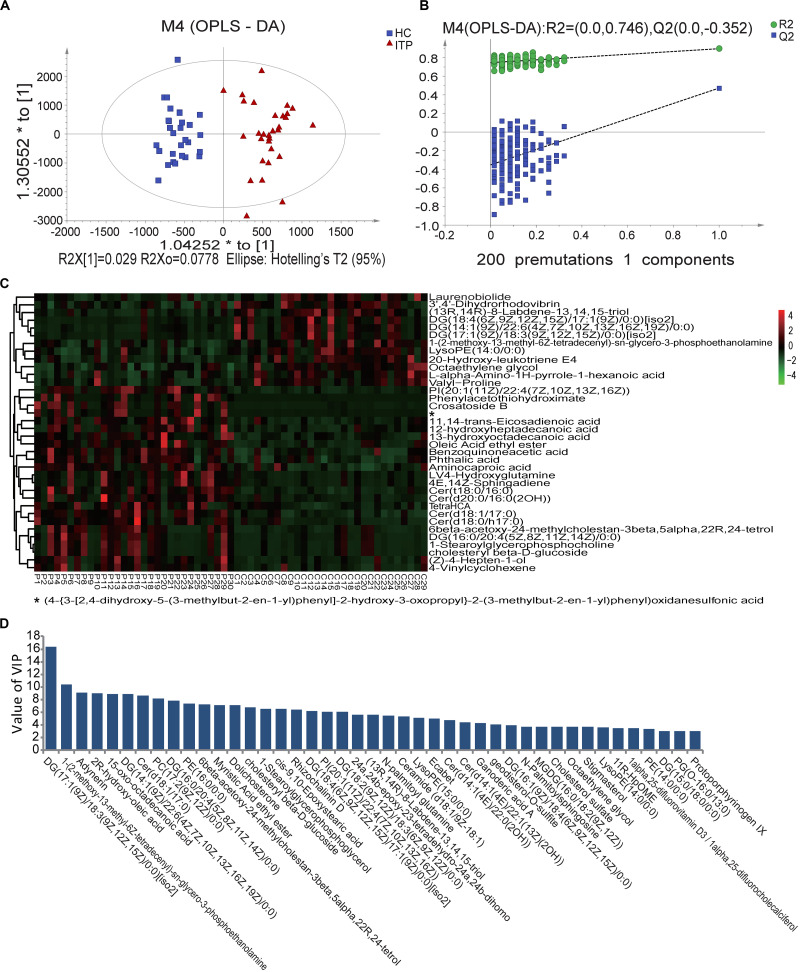
Score plots using UPLC/MS data of fecal metabolites. **(A)** OPLS-DA score plots from metabolite profiles of ITP and HC samples. **(B)** A 200 times permutation test of OPLS-DA model. **(C)** Heat maps of the significantly differential metabolites between different samples. Each sample is visualized in a single column and each metabolite is represented by a single row. Green colors indicate lower metabolite concentration while red colors show enhanced metabolite levels (see color scale). **(D)** Differential metabolites between HC and ITP samples with VIP values greater than 3.0.

Fecal metabolomes were analyzed using LC-MS under positive and negative ion modes. OPLS-DA resulted in separate clustering of the ITP and HC groups (Figure 2A), indicating that the fecal metabolomic profiles of these two groups were significantly different. The slope of the straight line was large and the intercept of Q2 was −0.352, indicating that OPLS-DA model was not over-fitting (Figure 2B). A total of 226 differential metabolites, with VIP values greater than 1.0 (*p* < 0.05) were identified, including 34 known classes and several unclassified compounds. The top ten classes ranked by mean relative abundances were fatty acyls (18.58%), prenol lipids (8.41%), steroids and steroid derivatives (7.94%), glycerophospholipids (7.52%), carboxylic acids (6.94%), sphingolipids (6.64%), steroids and steroid derivatives (6.19%), glycerolipids (5.75%), and organooxygen compounds (4.87%).

When the VIP threshold value was higher than 3.0, a total of 43 compounds were revealed on the OPLS-DA model (Figure 2D). Among them, the top ten metabolites ranked by VIP values were DG [17:1(9Z)/18:3(9Z,12Z,15Z)/0:0], 1-(2-methoxy-13-methyl-6Z-tetradecenyl)-sn-glycero-3-phosphoethanolamine, adynerin, 2R-hydroxy-oleic acid, 15-oxo-octadecanoic acid, DG [14:1(9Z)/22:6(4Z,7Z,10Z,13Z,16Z,19Z)/0:0], Cer (d18:1/17:0), PC [17:2(9Z,12Z)/0:0], DG [16:0/20:4(5Z,8Z,11Z,14Z)/0:0], and PE (16:0/0:0), indicating their great contributions to discriminating ITP patients and HCs in the OPLS-DA model.

To select more significantly different metabolites, from the total 226 fecal ones mentioned above, *p*_ajust_ < 0.05 was used as a threshold. This resulted in 36 metabolites (Figure 2C). Among them 61% were lipids and lipid-like molecules, with 67% enriched in ITP patients. These ITP-enriched metabolites mainly included four fatty acyls [aminocaproic acid, 13-hydroxyoctadecanoic acid, 12-hydroxyheptadecanoic acid, and (Z)-4-hepten-1-ol], four sphingolipids [Cer (t18:0/16:0), Cer (d18:1/17:0), Cer (d18:0/h17:0), and 4E,14Z-sphingadiene], two sterol lipids (6beta-acetoxy-24-methylcholestan-3beta,5alpha,22R,24-tetrol, cholesteryl beta-D-glucoside), two organooxygen compounds (crosatoside B, benzoquinoneacetic acid), two glycerophospholipids [PI (20:1(11Z)/22:4(7Z,10Z,13Z,16Z)), 1-stearoylglycerophosphocholine], phthalic acid, and L-4-hydroxyglutamine. Conversely, depleted fecal metabolites in ITP patients included three prenol lipids [(13R,14R)-8-Labdene-13,14,15-triol, 3′,4′-dihydrorhodovibrin and laurenobiolide], three glycerolipids [DG (18:4(6Z,9Z,12Z,15Z)/17:1(9Z)/0:0)[iso2], DG (17:1(9Z)/18:3(9Z,12Z,15Z)/0:0)[iso2] and DG (14:1(9Z)/22:6(4Z,7Z,10Z,13Z,16Z,19Z)/0:0)], two carboxylic acids and derivatives (valyl-proline and L-alpha-amino-1H-pyrrole-1-hexanoic acid), two glycerophospholipids [lysoPE (14:0/0:0) and 1-(2-methoxy-13-methyl-6Z-tetradecenyl)-sn-glycero-3-phosphoethanolamine].

### Certain ITP–Altered Gut Bacteria and Metabolites Show Good Diagnostic Potentials

**FIGURE 3:**
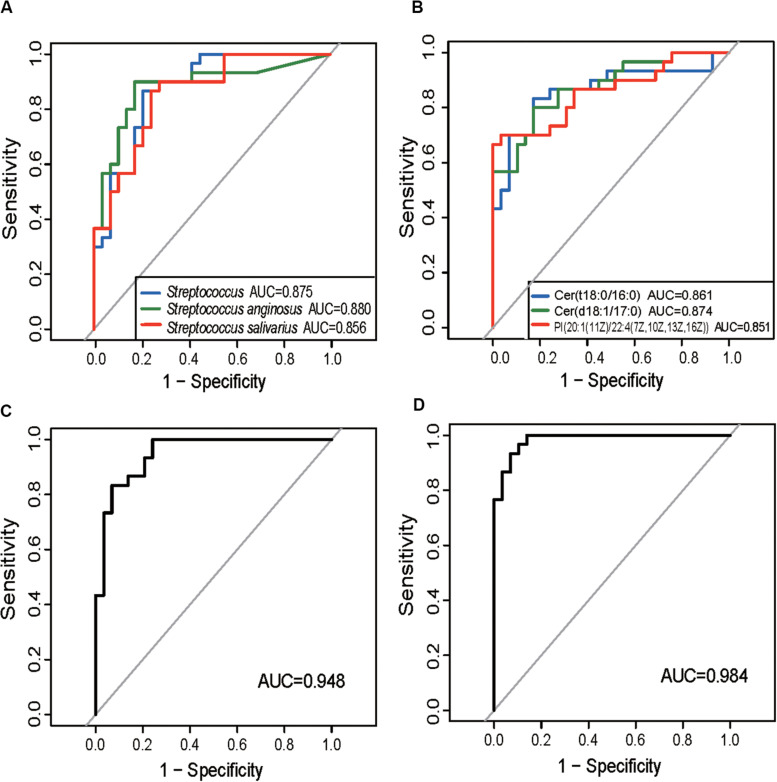
Prediction of ITP-enriched bacteria and metabolites. **(A)** Receiver-operating characteristic (ROC) plot for *Streptococcus*, *Streptococcus anginosus* and *Streptococcus salivarius*, areas under the parametric curve (AUC) value are 0.875, 0.880, and 0.856, respectively. **(B)** ROC plot for Cer (t18:0/16:0), Cer (d18:1/17:0), and PI [20:1(11Z)/22:4(7Z,10Z,13Z,16Z)], AUC are 0.861, 0.874, and 0.850, respectively. **(C,D)** The ROC analysis of the combination of *Weissella* and *Streptococcus anginosus*, or Cer (t18:0/16:0), Cer (d18:1/17:0), and 13-hydroxyoctadecanoic acid, with their corresponding AUC reaching 0.948 and 0.984, respectively.

The ITP-altered genera, species, and gut metabolites were evaluated for their potential diagnostic ability using ROC analysis. An AUC value higher than 0.85, which indicates good prediction, was used as the threshold during screening. Three microbial biomarkers *Streptococcus*, *S. anginosus* and *S. salivarius* with AUC values of 0.875, 0.88, and 0.856, respectively (Figure 3A) as well as three fecal metabolites (Cer (t18:0/16:0), Cer (d18:1/17:0), PI [20:1(11Z)/22:4(7Z,10Z,13Z,16Z)] (Figure 3B) with AUC values of 0.861, 0.874, and 0.85, respectively, were chosen as potential biomarkers for separating ITP patients from HCs. The combined variable ROC analysis showed that a better diagnosis could be obtained by combining *Weissella* and *S. anginosus*, or Cer (t18:0/16:0), Cer (d18:1/17:0), and 13-hydroxyoctadecanoic acid, as indicated by AUC values of 0.948 and 0.984, respectively (Figures 3C,D).

### ITP-Altered Gut Microbiota and Metabolites Closely Correlate With ITP Indicators

**FIGURE 4:**
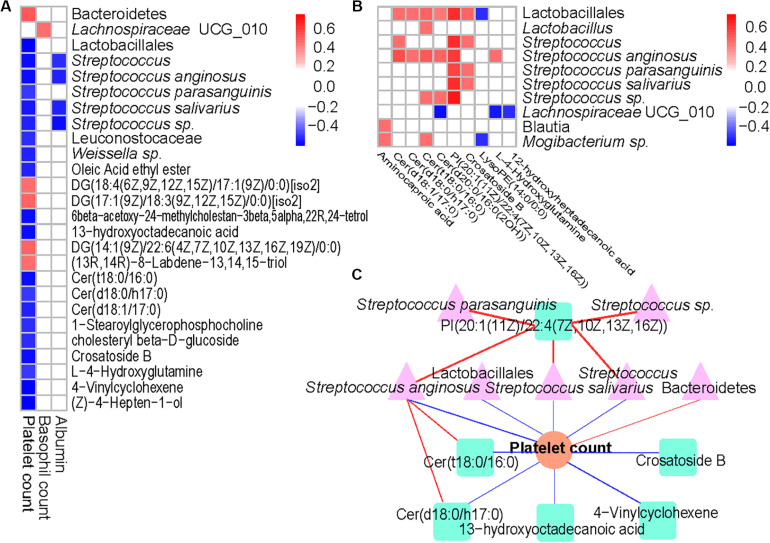
Associations between altered gut microbiome, metabolites, and hematological variables in ITP. **(A)** Association of ITP-altered fecal bacteria, metabolites with hematological variables. **(B)** The association of ITP-altered fecal bacteria with metabolites. Correlation coefficients (in absolute value) higher than 0.4 and *p* < 0.0001 were used as significance thresholds. **(C)** The association of ITP-altered gut microbiota and metabolites and platelet count. The pink color, light green, and orange represent bacteria, metabolites, and platelet count, respectively. Blue line represents negative correlation, while red line represents positive correlation.

Results from the Spearman’s correlation showed a predominantly positive correlation between levels of ITP-altered gut bacterial taxa and gut metabolites, at *p* < 0.0001. Among 31 pairs of correlations obtained, 26 showed a positive trend. Each one of *Lactobacillales* and their member *S. anginosus* showed a positive correlation with Cer (d18:1/17:0), Cer (d18:0/h17:0), Cer (t18:0/16:0), Cer [d20:0/16:0(2OH)] and PI [20:1(11Z)/22:4(7Z,10Z,13Z,16Z)]. In addition, high positive correlations of PI [20:1(11Z)/22:4(7Z,10Z,13Z,16Z)] with *Lactobacillales* (*p* = 2.30E-09), *Streptococcus* (*p* = 1.10E-09), *S. anginosus* (*p* = 2.78E-08), *S. parasanguinis* (*p* = 1.32E-07), *S. salivarius* (*p* = 4.01E-09) or *Streptococcus* sp. (*p* = 1.40E-05) were observed (Figure 4B).

On the other hand, platelet count was predominantly negatively correlated with ITP-altered gut microbiota and metabolites (Figure 4A). Among the 17 blood parameters included in the Spearman’s correlation analysis, only platelet, and basophil counts as well as albumin were correlated with gut microbiota and metabolites (*p* < 0.0001). However, platelet count was involved in nearly 83.33% of the total correlations. Lactobacillales and its members, including *Streptococcus* (*p* = 1.73E-06), *S. anginosus* (*p* = 2.00E-06) and *S. salivarius* (*p* = 8.95E-06) were highly negatively correlated with platelet counts, while *Bacteroidetes* (*p* = 4.92E-06) were highly positively correlated with platelet count. Meanwhile, lipids and lipid-like molecules Cer (t18:0/16:0) (*p* = 1.67E-06), 13-hydroxyoctadecanoic acid (*p* = 3.10E-06), Cer (d18:1/17:0) (*p* = 3.62E-06), Crosatoside B (*p* = 3.76E-06) and 4-Vinylcyclohexene (*p* = 9.11E-07) were highly negatively correlated with platelet count.

Then, we constructed a correlation network comprising ITP-altered bacterial taxa, metabolites and blood parameters using *p* < 10^–6^. Our results showed platelet count as the center of the network. Two important clusters of correlation networks for microbiota, metabolites and platelet count, except the positive correlation between *Bacteroidetes* and platelet count mentioned above were evident (Figure 4C). With regards to platelet count, one cluster comprised *S. anginosus* with Cer (t18:0/16:0) and/or Cer (d18:0/h17:0), while the other had PI [20:1(11Z)/22:4(7Z,10Z,13Z,16Z)] with *Streptococcus* and/or *S. anginosus* and/or *S. salivarius* and with platelet count.

### Predicted Metagenomes of Gut Microbiota Vary Between ITP Patients to HCs

**FIGURE 5:**
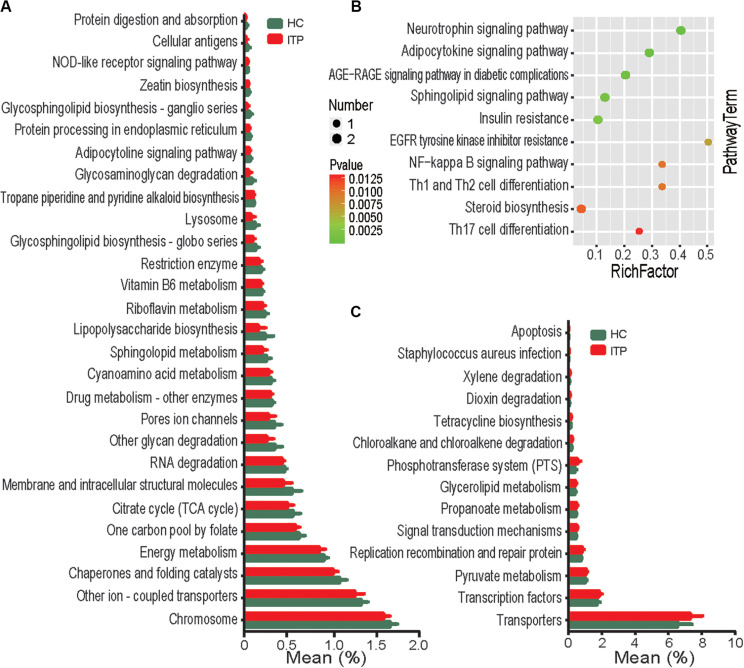
Functional analysis of predicted metagenomes. **(A)** PICRUSt results of downregulated metabolic pathways in the gut microbiome of ITP patients. **(B)** Top 10 enriched metabolic pathways between the HC and ITP groups. **(C)** PICRUSt results of elevated metabolic pathways in the gut microbiome of ITP patients.

To assess potential differences in microbiota functions between HCs and ITP patients, we predicted functional gene compositions in the metagenomes based on Bacterial OTU tables at PICRUSt, a bioinformatics tool with evolutionary modeling and reference genomic databases. Figure 5A shows a total of 28 pathways related to bacterial functions, including xylene degradation, dioxin degradation, and signal transduction mechanisms, were found weakened in gut microbiota of ITP patients. However, 14 pathways, including those related to NOD-like receptor signaling, protein digestion and absorption, glycosphingolipid biosynthesis-ganglio series, glycosaminoglycan degradation and other glycan degradation were strengthened in gut microbiota of ITP patients (Figure 5C).

### ITP-Altered Gut Metabolites Were Involved in Inflammation Pathways

To explore the potential contribution of known metabolites to ITP, 226 ITP-altered metabolites were submitted to the KEGG website for pathway analysis. Our results show that ITP-enriched metabolites were mainly related to pathways such as neurotrophin signaling pathway, Th1, Th2, and Th17 cell differentiation (Figure 5B), indicating that enrichment of these metabolites may be potentially linked to inflammation.

## Discussion

Increasing research efforts have focused on the relationship between gut microbiota and autoimmune diseases as well as mechanisms involved in their interactions. So far, alterations of gut microbiota in more than 20 types of autoimmune diseases, including type 1 diabetes, multiple sclerosis, rheumatoid arthritis, ankylosing spondylitis, autoimmune hepatitis, and Crohn’s disease, have been reported relative to HCs (Alkanani et al., 2015; Zhang et al., 2015; Jangi et al., 2016; Mottawea et al., 2016; Wen et al., 2017; Wei et al., 2019). However, to the best of our knowledge, there has been no report on gut microbiota in patients with ITP. The findings of this study showed that the alterations of ITP gut microbiota were mainly due to enrichment of *Lactobacillus*, *Streptococcus* and their members, as well as depletion of *Bacteroides* and their members. In addition, fecal metabolomes of ITP patients comprised ITP-enriched Cer (t18:0/16:0) and the ITP-depleted lysoPE (14:0/0:0) and were significantly different from those of HCs. Several ITP-altered bacteria such as *S. anginosus* and ITP-altered metabolites such as Cer (t18:0/16:0) had good diagnosis potential and intensively correlated with platelet count. Moreover, predicted metagenomes of gut microbiota were significantly different from ITP patients relative to HCs. Finally, KEGG analysis showed that several altered metabolites were intensively involved in inflammatory pathways. Overall, these results may contribute to research in pathogenesis research, diagnosis and treatment of ITP.

Alterations of gut microbiota in ITP patients showed similarity to some but also varied from other known autoimmune diseases. Particularly, our results showed that *Actinomycetaceae*, *Lactobacillus*, and *Streptococcus* were enriched, while Bacteroidetes was depleted in ITP patients. In patients with Crohn’s disease, gut Bacteroidales were depleted, while Proteobacteria and *Veillonella* were enriched (Mottawea et al., 2016). In patients with rheumatoid arthritis, *Lactobacillus salivarius* were enriched, with no observation of Bacteroidetes depletion (Zhang et al., 2015). Patients with primary biliary cirrhosis were found to show enrichment of *Actinobacteria*, *Lactobacillales*, *Streptococcus*, *Klebsiella* and *Veillonella* (Lv et al., 2016). Other studies have reported enrichment of Actinobacteria in patients with ankylosing spondylitis, and depletion of *Fusobacteria* and *Verrucomicrobia* (Wen et al., 2017) while those with type 1 diabetes, showed a combination of an increased relative abundance of *Bacteroidetes* together with decreased levels of *Firmicutes* regardless of geographical location, which is contrary to their alterations in ITP (Siljander et al., 2019). Overall, we infer that the alterations in gut microbiota of ITP patients possess several special characteristics that make them critical to the occurrence and development of the condition. This is despite not listing all the differences in gut microbiota between all known autoimmune diseases and ITP, and the fact that accuracy of the comparisons is limited by possible differences in technology and methods between different studies.

Some bacteria, similar to ITP-enriched gut taxa, play important roles in immunity and autoimmunity. First, our results showed *Lactobacillus* and *Streptococcus* (including *S. anginosus, Streptococcus* sp., *S. parasanguinis*, and *S. salivarius*) were enriched in ITP patients. The Lactobacillaceae family and its most abundant genus *Lactobacillus* in ITP patients were significantly higher than in HCs, consistent with the results in patients with severe fever with thrombocytopenia syndrome (SFTS) (Xu et al., 2018). Recent studies revealed regulation of intestinal microbiota in autoimmune arthritis by promoting differentiation and migration of T follicular helper (Tfh) cells (Block et al., 2016; Teng et al., 2016). *Lactobacillus* was demonstrated to enhance antigen-specific IgA secretion and induce Tfh cells in mice (Satoshi et al., 2018). Most importantly, the involvement of Tfh cells in the pathogenesis of ITP has been well documented (Audia et al., 2017). Therefore, it is speculated that *lactobacillus* might indeed play a key role in the progression of ITP. However, further validation is needed. Second, *S. anginosus* is increasingly recognized as an opportunistic pathogen that can cause diseases such as bloodstream infections, bacteremia and invasive pyogenic infections. *S. anginosus* strain NCTC10713T, showed clear β-hemolysis on blood agar plates. In addition, *Streptolysin S* produced from this strain exhibited a cytotoxic potential similar to that produced by *Streptococcus pyogenes*. The result was a peptide hemolysin that exhibits hemolytic activity on erythrocytes as well as cytotoxic activity in cell culture lines *in vitro* and *in vivo* using a mouse model (Tabata et al., 2019). Autoimmune disorders, induced by some other members of *Streptococcus* except *S. anginosus*, have also been reported. For example, group G *Streptococcu*s induced an autoimmune carditis mediated by IL-17A and interferon gamma in the Lewis rat model of rheumatic heart disease (Sikder et al., 2018). A small fraction of children with *S. pyogenes* infections was reported to develop Sydenham’s chorea or pediatric autoimmune neuropsychiatric disorders (Cutforth et al., 2016). Results of the current study showed that *S. anginosus* not only increased 35-fold in its relative abundance from ITP patients to HCs, but was also highly negatively correlated to platelet count. Overall, these results demonstrate that ITP-enriched bacteria, such as *S. anginosus*, may play important roles in ITP occurrence.

Besides, our results showed depletion of Bacteroidetes and increase of Firmicutes/Bacteroidetes ratio in ITP patients relative to HCs. Members of Bacteroidetes, mainly Gram-negative bacteria, mostly colonize the distal gut. They can ferment indigestible polysaccharides, thus producing short-chain fatty acids thereby providing energy for the host (Gibiino et al., 2018). Moreover, they contribute play a role in development and maintenance of host immunity. Deletion of the TLR2 gene (the main receptor of lipopolysaccharide from cell wall of Gram-negative bacteria, including Bacteroidetes) decreased platelet production and numbers (Kovuru et al., 2019). Besides, Firmicutes/Bacteroidetes ratio is considered a representative of health status and may reflect eubiosis of the gastrointestinal tract (Qin et al., 2010).

Gut metabolites can be an intermediary of the crosstalk between gut microbiota and their host. Recently, analysis of fecal metabolomes has unraveled a correlation between metabolic phenotype changes and gut microbiota perturbations during the development of autoimmune conditions, including IBS and Crohn’s disease (Jansson et al., 2009; Le Gall et al., 2011). Our results showed a significant difference in fecal metabolic profiles between ITP and those of normal controls. Then, we constructed a correlation network comprising ITP-altered bacterial taxa, metabolites and blood parameters. Our results showed a predominantly correlation between microbiota, metabolites, and platelet count, which suggested the intestinal flora and its metabolites may play a key role in the development of ITP. In addition, PICRUSt analysis revealed that the enriched KEGG pathways of gut microbiota in ITP mostly focused on lipid metabolism. Previous studies demonstrated important role of abnormal lipid metabolism in autoimmune diseases, such as diabetes and multiple sclerosis (Kim, 2017; Kurz et al., 2018). Therefore, we proposed possible involvement of lipids and lipid-like molecules in the onset and development of ITP. Meanwhile, our KEGG-based pathway analysis results showed that ITP-altered metabolites significantly located in Th1/Th2 and Th17 cell differentiation, which is closely related to ITP pathogenesis (Wang et al., 2005; Rocha et al., 2011).

The role of gut microbiota during disease diagnosis has attracted increasing attention. So far, no “gold standard” can be used as a reliable diagnosis for ITP. A presumptive test for this condition is mainly based on exclusion, which largely depends on clinical expertise and observations. A detailed history and examination are essential to ensure elimination of thrombocytopenia from other causes of thrombocytopenia, such as presence of lymphadenopathy, and help identify potential secondary causes (Ejaz and Radia, 2019). Our results showed that several gut bacteria and metabolites, such as *S. anginosus* and Cer (t18:0/16:0), had great potential as invasive diagnostic biomarkers for ITP. Ceramides have also been described as specific disease biomarkers in other autoimmune diseases, including multiple sclerosis and rheumatoid arthritis (Kosinska et al., 2014; Kurz et al., 2018). However, little is known regarding the potential of *S. anginosus* for diagnosis of other autoimmune diseases, besides ITP. Combinations of several ITP-altered gut bacteria and/or metabolites from our results, can not only improve ability, but also avoid repetition of a single biomarker for diagnosing other autoimmune diseases. However, the effectiveness of the diagnostic capabilities of these biomarkers will need to be verified using larger studies in future. Furthermore, the excellent diagnosis potential of gut bacteria and metabolites indicate that they are also critical to the occurrence or development of ITP.

Although our investigations attempt to provide a comprehensive insight into the potential contribution of the gut microbiome in ITP, this study has several limitations that must be addressed in future studies. Firstly, this was a single-center, cross-sectional study involving a small number of samples. Secondly, although ITP patients were matched for age, sex, and BMI in the analysis, the results may be influenced by other confounding effects, such as dietary factors. Thirdly, the study does not include animal experiments or researches regarding the possible mechanism linking gut microbiota and metabolism to the development of ITP. Therefore, further studies are needed with larger data set of patients and animal experiments to explore the potential causal mechanisms between gut microbiota and ITP.

## Conclusion

In summary, our study identified alterations of gut metabolome, particularly in lipids, elucidated dysbiosis of gut microbiota characterized by enrichment of *Streptococcus*, *Lactobacillus*, and depletion of *Bacteroidetes* in ITP patients. Moreover, the findings showed that some ITP-altered gut bacteria and metabolites have great potential for diagnosis of ITP. This is based on an evidently positive correlation of these parameters with platelet count, suggesting that they may also play a role in ITP pathogenesis. Identifying the key players from gut microbiota and illustrating their roles and mechanism of action in ITP occurrence and development are further avenues for future research.

## Data Availability Statement

The datasets presented in this study can be found in online repositories. The names of the repository/repositories and accession number(s) can be found in the article/supplementary material.

## Ethics Statement

The studies involving human participants were reviewed and approved by the ethics committee of the First Affiliated Hospital of Zhejiang University (reference number: 2018-42). The patients/participants provided their written informed consent to participate in this study.

## Author Contributions

WQ conceived and designed this study. XZ, SG, and LL performed the experiments, collected and analyzed the data, and wrote the manuscript. LY, YX, DZ, YC, RY, HJ, and YL collected and analyzed the data. All authors contributed to the article and approved the submitted version.

## Conflict of Interest

The authors declare that the research was conducted in the absence of any commercial or financial relationships that could be construed as a potential conflict of interest.

## Footnotes

1 http://qiime.org/

2 http://drive5.com/uparse/

3 http://rdp.cme.msu.edu/

4 http://www.R-project.org/

5 https://pan.baidu.com/s/1Gv0fLnhriJMsM6qj3rUGdg

## Abbreviations

ALT: alanine aminotransferase
AST: aspartate aminotransferase
AUC: area under curve
HCs: healthy controls
ITP: immune thrombocytopenia
LC-MS: liquid chromatography-mass spectrometry
OPLS-DA: orthogonal partial least squares discriminant analysis
OTUs: operational taxonomic units
PCoA: principal coordinates analysis
ROC: receiver operating characteristic
VIP: variable importance in the projection.
